# Phloem transport capacity of transgenic rice T1c-19 (Cry1C*) under several potassium fertilizer levels

**DOI:** 10.1371/journal.pone.0195058

**Published:** 2018-03-29

**Authors:** Lin Ling, Yang Jiang, Jiao Jing Meng, Li Ming Cai, Gui Cou Cao

**Affiliations:** Ministry of Agriculture Key Laboratory of Crop Physiology, Ecology and Cultivation (The Middle Reaches of Yangtze River), Huazhong Agricultural University, Wuhan, Hubei, China; University of Delhi, INDIA

## Abstract

Genetic modification of Cry-proteins from *Bacillus thuringiensis (Bt)* is a common practice in economically important crops to improve insecticide resistance and reduce the use of pesticides. However, introduction of these genes can have unintended side effects, which should be closely monitored for effective breeding and crop management. To determine the potential cause of these negative effects, we explored assimilate partitioning in the transgenic *Bt* rice line T1c-19 (Cry1C*), which was compared with that of its wild-type counterpart Minghui 63 (MH63) under different potassium fertilization application treatment conditions. In a pot experiment, 0, 0.4, and 0.6 g K_2_O was applied per kg of dry soil to determine the phloem transport characteristics of the two rice lines. We used a variety of assessment indicators ranging from morphological to physiological aspects, including the number of large and small vascular bundles in the neck internode at the heading stage, the diameter and bleeding intensity of the neck internode at the filling stage, and the content and apparent ratio of transferred non-structural carbohydrates (NSC) in the culm and sheath from the heading to maturing stages. The K utilization and grain yield at the maturing stage were also concerned. Results presented that the mean setting rate and grain yield of T1c-19 (Cry1C*) decreased by 22.3% and 26.2% compared to those in MH63, respectively. Compared to MH63, the K concentration and accumulation were significantly higher in the culms and leaves, but significantly lower in grain of T1c-19 (Cry1C*). T1c-19 (Cry1C*) had less apparent NSC efflux in the culm and sheath, fewer small vascular bundles, and a smaller diameter and bleeding intensity of the neck internode than MH63. In addition, linear correlation analysis indicated that there were positive correlations among grain yield, setting rate, the apparent NSC efflux in the culm and sheath, number of small vascular bundles, and the neck internode diameter and bleeding intensity. These unintended effects may directly or indirectly be caused by insertion of exogenous *Bt* (Cry1C*) gene, which should be further considered in the future breeding of transgenic crops.

## Introduction

Genetically modified crop plants engineered for pest resistance represent a promising tool to help decrease the amount of chemical pesticides that are applied in agriculture [1–3]. Cry-proteins from *Bacillus thuringiensis* (*Bt*) are by far the most common insecticidal proteins that have been engineered into a large number of plant species, including corn, cotton, potato, tomato, eggplant, and rice [4, 5]. However, unintended effects in transgenic crops widely occur in the production process. Thus, avoiding further unintended effects is the primary goal for breeding experts.

The unexpected effects of *Bt* gene expression in rice include decreased stem and root height, reduction in grains per panicle, and a low grain filling percentage. In particular, a low grain filling percentage can lead to yield loss, which has been reported in transgenic rice expressing different *Bt* genes [6–9]. Lynch et al. [10] showed that transgenic *Bt* rice plants were smaller, had a later flowering time, and lower fertility compared with their non-transformed wild types. In addition, 16 amino acids were found to be present in lower amounts in the leaves of *Bt* rice when compared to those detected in conventional rice [11, 12] Moreover, the leaf stomatal conductance and light saturation net photosynthetic rate in *Bt* rice were reported to be low at the tillering stage, jointing–booting stage, and milky stage [13]. However, the cause of these effects is still unclear. The assimilation process is one of the most important factors contributing to the yield of rice. Assimilation products are transported mainly through vascular bundles distributed throughout the leaves, stem, and roots of rice plants [14, 15]. Therefore, the transportation process is of great importance for plant growth, especially during the filling period in the late stage of development.

Potassium (K) is confirmed to be the most important and abundant cation in plant and constitutes 2–10% of plant dry weight [16]. It highly involves in activating enzyme, neutralizing anion. Besides, K^+^ plays crucial role in membrane transport and osmoregulation [17]. Thus, it directly influences the phloem transportation, the efficiency of photosynthesis, and the transportation and/or allocation of assimilation products (such as sugar) in rice [18–21]. Namely, in rice, the K supply can affect the leaf area, photosynthetic rate, and the synthesis and transport of carbohydrates, as well as the transportation process under different field nutrient conditions, and this function cannot be compensated by the other two necessary elements nitrogen and phosphorus.

Previous studies have demonstrated clear differences in nitrogen, phosphate, and silicon contents between transgenic and conventional rice [11, 12]. The absorption and utilization of K in *Bt* cotton were also found to be different from those measured in conventional cotton lines [22]. However, few studies have investigated the influence of the *Bt* gene on plant physiological characteristics, particularly with respect to the absorption and utilization of K and the subsequent consequences, including phloem transportation capacity.

There is currently no information available as to the influence of any unintended effects caused by insertion of the *Bt* gene on K absorption and utilization or the transportation of assimilation products in plants. Toward this end, in this study, we explored the K utilization and assimilate transportation capacity of the T1c-19 rice variety, which expresses the *Bt*-Cry1C* gene, which was compared to its non-transgenic counterpart Minghui 63 (MH63), and elite indica rice cultivar that is often used as an important restorer. These findings should provide a valuable scientific foundation for breeding scientists to improve transgenic rice varieties.

## Materials and methods

### Plant materials

The transgenic *Bt* rice line T1c-19 applied in this experiment was provided by the National Key Laboratory of Crop Genetic Improvement, Wuhan, China. T1c-19 was obtained after transforming a modified Cry1C* gene into MH63 [23], which was selected as a highly insect-resistant line in the field among 120 independently transformed plants in light of its especially high content of Cry1C* protein. We selected this line in particular, since the Cry1C* protein is considered one of the most promising *Bt* proteins for future transgenic crop breeding, because of its higher toxicity at lower content compared with Cry2A*, *Cry1Ab/Ac*, and other *Bt* proteins conferring insect resistance.

### Experimental design

The pot experiment was conducted in a screen house at Huazhong Agricultural University, Wuhan (29°58′N 113°53′E), China. Seeds from each rice line were sown on June 3 and transplanted into 30 cm × 30 cm pots with 10 kg of soil June 18. The soil of each pot was fertilized with 0.4 g N and 0.2 g P_2_O_5_ as a base fertilizer, and 0 g, 0.4 g, and 0.6 g K_2_O were used as the three supplemental K levels for the duration of the experiment, designated K0, K1, and K2, respectively. In addition, 50% base fertilizer, 30% tillering fertilizer, and 20% head manure was used as the nitrogen source. One-time application of NaH_2_PO_4_ was used as phosphate fertilizer. Finally, 50% K fertilizer was applied at the base stage and then 50% was again applied at the heading stage.

Soil for the pot experiments was collected from the top layer (0–20 cm) of a conventional paddy field at Huazhong Agricultural University, Wuhan, Hubei Province, China. Any residual roots were removed from the soil before it was air-dried at room temperature, mixed with dinas, and homogenized through a sieve for potting. This soil contained 13.11 g kg^−1^ of organic matter, 1.02 g kg^−1^ of total N, and 97 mg kg^−1^ of available K, with a pH (H_2_O) of 6.53.

Five replicates of each treatment were established for the two rice lines, representing a total of 210 pots that were randomly placed in the screen house. During the plant growth period, water was maintained at approximately 1 cm above the soil surface, and pest and disease damage were also strictly controlled.

### Sampling and data collection

#### Collection of assimilate transportation indicators

The number of large and small panicle neck vascular bundles was counted at the full heading stage. Five panicles from every pot were taken for measurement. Samples were taken between the neck panicle node and 3 cm below the node and then preserved in 60% ethanol before analysis. The diameter and bleeding intensity of the panicle neck node of five similar panicles were measured at the filling stage.

#### Panicle neck vascular bundle number

Five panicle neck nodes from plants in one pot were soaked in 60% ethanol before they were processed to obtain slices from the middle section of the tissue. For each replicate, five slices were collected and stained with safranin. After staining, large and small panicle neck vascular bundles were counted using an optical microscope.

#### Diameter and bleeding intensity of the panicle neck node

The maximum diameter of the middle of the selected panicle neck node was measured using Vernier calipers.

The collection of bleeding sap was performed for 24 h after 16:00 using a bleeding sap tube filled with weighed absorbent cotton. During the collection period, the cotton was firmly covering the panicle neck node. The difference in the weight of the cotton before and after bleeding was divided by 24 to calculate the bleeding intensity per hour (mg h^-1^).

#### Determination of nonstructural carbohydrates (NSC) in the stem sheaths of rice

The sampling of stem sheaths included five replicates from each pot at the full heading, filling, and maturing stages. Stem sheaths were ground and sieved for the assay after reaching a constant weight after drying at 70°C.

The NSC (including water soluble sugar and starch) concentration (%) was tested by anthrone colorimetry [24], and the NSC content and its apparent output were calculated using the respective equations below:

NSC content (g/pot) in the stem sheath at the full heading stage, filling stage, or maturing stage = NSC concentration × dry weight of the stem sheath at the full heading stage, filling stage, or maturing stage

NSC apparent output in the stem sheath (g/pot) = NSC content at the full heading stage–NSC content at the maturing stage

#### Assessment of K concentration in rice plants

The aboveground material from the rice plants was separated into the panicle, stem, and leaf. Each part was dried at 70°C until it maintained a constant weight. The samples were then ground in a ball mill and passed through a sieve before analysis. The K concentration (mg g^-1^) was tested at the maturing stage using flame photometry after digesting the prepared sample in concentrated H_2_SO_4_–H_2_O_2._

K accumulation in the stems and leaves or grain (g/pot) = K concentration of stem and leaf or grain × dry weight of stems and leaves or grain.

Total K accumulation of aboveground organs of the rice plant (g/pot) = K accumulation of stem and leaf + K accumulation of the grain.

#### Yield

The panicles were obtained at the mature stage for yield assessment. The number of filled and unfilled spikelets was separated using the water-selection method. The setting rate was expressed using the ratio of filled grain to the total grain.

### Data analysis

SAS 9.1 software (SAS Institute, Inc., Cary, NC, USA) was used for analysis of variance (ANOVA) of data obtained in this experiment. Data are expressed as the means ± standard deviation (SD, n = 5). Differences in the means were considered to be statistically significant at α = 0.05.

## Results

### Comparison of grain yield and yield components in T1c-19 (Cry1C*) and MH63 rice

There were no significant differences between T1c-19 (Cry1C*) and MH63 rice with respect to the number of panicles per pot, spikelets per panicle, spikelets per pot, or 1,000-grain weight. However, the setting rate of T1c-19 (Cry1C*) was 15.7%, 25.3%, and 25.8% lower than that in MH63 in the zero K (K0), normal K (K1) and high K (K2) treatments, respectively (Table 1). In other words, the variance of the setting rate increased in proportion to the input of K fertilizer. The grain yield of each pot of T1c-19 (Cry1C*) decreased by 28.7%, 25%, and 25% as compared with that of MH63 for the K0, K1 and K2 treatment, respectively. Thus, the reduction of grain yield at different K levels might be caused by the lower setting rate.

**Table 1:** Yield and components of rice grown in different potassium levels.

Potassium Level	Line	Panicle (No. per pot)	Spikelet (No. per panicle)	Spikelet (No. per pot)	Setting rate (%)	1,000-grain weight (g)	Grain yield (g per pot)
K0	MH63(Cry1C*)	61 a	96 a	5863 a	65.4 b	24.6 a	97 b
	MH63	60 a	102 a	6067 a	77.6 a	26.8 a	136 a
K1	MH63(Cry1C*)	54 a	102 a	5510 a	55.3 b	26.7 a	90 b
	MH63	57 a	106 a	6038 a	74.0 a	24.4 a	120 a
K2	MH63(Cry1C*)	59 a	97 a	5721 a	58.2 b	27.0 a	93 b
	MH63	61 a	101 a	6111 a	78.4 a	25.8 a	124 a

Values followed by different letters are significantly different between the two rice lines grown in the same potassium levels at p<0.05.

### Comparison of K absorption and utilization in T1c-19 (Cry1C*) and MH63

Total K accumulation in aboveground plant organs in T1c-19 (Cry1C*) was remarkably higher than that in the MH63 wild-type for the K0 and K1 treatments, but there were no significant differences in K2 treatment. Thus, overall K accumulation in T1c-19 (Cry1C*) was not less than that of MH63. In addition, for the three K levels, the weight, K concentration (except for the K2 level), and the K accumulation in the stems and leaves were higher than that those measured in the MH63 rice. However, this pattern was reversed in the grain, with lower values for T1c-19 (Cry1C*) than in MH63 (Table 2). K accumulation in T1c-19 (Cry1C*) stems and leaves was 28.0%, 23.9%, and 15.8% higher than those in MH63; By contrast, in grains, K accumulation was 25.0%, 33.0%, and 42.0% lower in T1c-19 (Cry1C*) than those in MH63. Therefore, T1c-19 (Cry1C*) stems and leaves accumulated more K, but the grains accumulated less, which partly because of differences in the transportation process from the stems to leaves to grains.

**Table 2:** The potassium concentration and accumulation in rice grown in different potassium levels.

Potassium level	Line	Dry weight (g per pot)		K concentration (mg g^-1^)		K accumulation (g per pot)		Total K accumulation in above-ground plant parts (g per pot)
		Stems and leaves	Grain	Stems and leaves	Grain	Stems and leaves	Grain	
K0	MH63(Cry1C*)	164 a	82.0 b	14.7 a	5.07 b	2.42 a	0.42 b	2.84 a
	MH63	151 b	102.9 a	12.6 b	5.46 a	1.89 b	0.56 a	2.45 b
K1	MH63(Cry1C*)	170 a	72.3 b	17.6 a	4.70 b	3.01 a	0.34 b	3.35 a
	MH63	153 b	99.8 a	15.9 b	5.10 a	2.43 b	0.51 a	2.94 b
K2	MH63(Cry1C*)	157 a	69.1 b	17.8 a	4.18 b	2.79 a	0.29 b	3.08 a
	MH63	141 b	100.5 a	17.1 a	4.95 a	2.41 b	0.50 a	2.91 a

Values followed by different letters are significantly different between the two rice lines grown in the same potassium levels at p<0.05.

### Phloem transportation capacity T1c-19 (Cry1C*) compared with MH63

#### Neck panicle node morphological structure and bleeding intensity

There were no significant differences in the number of large vascular bundles in the neck panicle node of T1c-19 (Cry1C*) and MH63 plants at the full heading stage presented no significant differences, whereas the number of small vascular bundles, neck panicle node diameter, and bleeding intensity were all lower in T1c-19 (Cry1C*) than those in MH63 (Table 3). The average difference of each index between the *Bt* line and MH63 at the three K levels was 13.0%, 6.5%, and 35.8%, respectively.

**Table 3:** The number of vascular bundles, diameter, and bleeding intensity of the neck internode in rice grown in different potassium levels.

Potassium level	Line	HD		FS	
		Large vascular bundle (No culm^-1^)	Small vascular bundle (No culm^-1^)	Diameter (mm)	Bleeding intensity (mg h^-1^)
K0	MH63(Cry1C*)	13.7 a	21.4 b	2.01 b	0.50 b
	MH63	14.7 a	25.2 a	2.22 a	0.85 a
K1	MH63(Cry1C*)	14.5 a	23.6 b	2.06 b	0.62 b
	MH63	15.1 a	25.2 a	2.18 a	1.14 a
K2	MH63(Cry1C*)	12.9 a	21.2 b	1.94 b	0.71 b
	MH63	13.5 a	25.7 a	2.03 a	0.86 a

HD: Heading stage; FS: Filling stage. Values followed by different letters are significantly different between the two rice lines grown in the same potassium levels at p<0.05.

#### Nonstructural carbohydrates (NSC) in rice stem sheaths

There were no significant differences in the dry weight of stem sheaths, NSC concentration, or NSC content between T1c-19 (Cry1C*) and MH63 in the three K levels at the heading stage. However, these indicators were significantly higher in T1c-19 (Cry1C*) at both the filling and maturing stages (Table 4). Thus, the apparent NSC output in the stem sheaths of T1c-19 (Cry1C*) indicated that the amount of NSC transported from the stems to the leaves was 34.8%, 24.4%, and 28.3% less NSC than that in MH63 at the three in all K levels used in this experiment.

**Table 4:** Concentration, content, and apparent efflux of NSC in rice culm and sheaths grown in different potassium levels.

Potassium level	Line	Dry weight (g per pot)			Concentration of NSC (%)			Content of NSC (g per pot)			Apparent efflux of NSC (g per pot)
		HD	FS	MS	HD	FS	MS	HD	FS	MS	
K0	MH63(Cry1C*)	245 a	186 a	167 a	33.7 a	16.4 a	23.5 a	82.6 a	30.5 a	39.2 a	43.4 b
	MH63	253 a	162 b	145 b	35.1 a	8.3 b	15.3 b	88.8 a	13.4 b	22.2 b	66.6 a
K1	MH63(Cry1C*)	269 a	185 a	173 a	35.5 a	20.1 a	22.8 a	95.5 a	37.2 a	39.4 a	56.1 b
	MH63	263 a	157 b	143 b	36.4 a	9.9 b	15.0 b	95.7 a	15.5 b	21.5 b	74.2 a
K2	MH63(Cry1C*)	257 a	182 a	176 a	37.8 a	20.3 a	22.2 a	97.1 a	36.9 a	39.1 a	58.0 b
	MH63	264 a	158 b	149 b	38.7 a	12.7 b	14.3 b	102.2 a	20.1 b	21.3 b	80.9 a

NSC: The non-structural carbohydrates; HD: Heading stage; FS: Filling stage; MS: Maturing stage. Values followed by different letters are significantly different between the two rice lines grown in the same potassium levels at p<0.05.

### Correlation analysis of sink and flow characteristics

Grain yield and flow characteristics excluding the number of large vascular bundles in the stem sheath, showed significantly positive correlations. In addition, the setting rate, NSC apparent efflux, small vascular bundle numbers, diameter of neck panicle node, and bleeding intensity were also positively correlated. Among the flow characteristics, the NSC apparent efflux was significantly related to the number of small vascular bundles in the neck panicle node and its bleeding intensity. Similar results were found between the neck panicle node diameter and small vascular bundle number, as well as the neck panicle node diameter, node bleeding intensity, large and small vascular bundle numbers, large vascular bundle numbers and diameter of the neck panicle node (Table 5).

**Table 5:** The correlations between sink and flow characteristics.

	NLVB_NI_	NSVB_NI_	D_NI_	BI_NI_	AOT_NSC_	Setting rate	1,000-grain weight
NSVB_NI_	0.52 *						
D_NI_	0.51 *	0.61 **					
BI_NI_	0.25	0.57 *	0.53 *				
AOT_NSC_	0.25	0.55 *	0.35	0.74 **			
Setting rate	0.17	0.63 **	0.52 *	0.57 *	0.66 **		
1000-grain weight	-0.07	-0.16	-0.17	-0.13	-0.22	-0.23	
Grain yield	0.16	0.71 **	0.58 **	0.61 **	0.67 **	0.87 **	-0.04

The data are correlation coefficients, “*” (“**”) mean the values are significantly different at p<0.05 (0.01). NLVB_NI_: Number of large vascular bundles in the neck internode; NSVB_NI_: Number of small vascular bundles in the neck internode; D_NI_: Diameter of the neck internode; BI_NI_: Bleeding intensity of the neck internode; AOT_NSC_: Apparent efflux of NSC in the culm and sheath.

## Discussion

Several *Bt* cotton varieties are sensitive to K deficiency, producing low dry weight and K uptake under low K application [22, 25, 26]. Analysis of the allocation of 12 mineral elements in the bivalent transgenic cotton line *Bt + CpTI* indicated that the K content in the roots, stems, and leaves was lower than that in the same tissues of non-*Bt* cotton, especially in the leaves [27]. However, in our study, the K accumulation in the aboveground organs of *Bt* rice was not less than that in the non-*Bt* rice line, and in the K0 condition, the K accumulation in *Bt* rice appeared to be higher than that in non-*Bt* rice. Furthermore, when compared to the non-*Bt* rice line, K accumulation in *Bt* rice was higher in the stems and leaves but lower in the grains. This difference may be caused by changes in K transportation from the stem and leaf to the grain. Yukui et al. [27] proposed that the reduced K accumulation in *Bt* cotton could be caused by changes in the binding protein functions responsible for mineral element absorption and allocation. These differences may arise from a higher sensitivity to K in cotton or from the different *Bt* genes that were inserted into the cotton lines; however, no similar study has been conducted in *Bt* rice, and thus the mechanism of K sensitivity in *Bt* rice requires further study.

K allocation may be correlated with rice yield, while the cause and effect relationship between K and rice development remains unknown. The difference in K distribution between the stems, leaves, and grain in *Bt* and non-*Bt* rice could lead to the yield variance observed in the present study. Another possibility is that the reduction in yield in T1c-19 (Cry1C*) was affected by the K distribution and low setting rate, which caused the decrease in spikelet dry matter and reduced K accumulation. Similarly, Wang et al. [8] suggested that the reduction in T1c-19 (Cry1C*) yield was due to a lower setting rate, which might be derived from a drop in growth hormones in the spikelets.

The filling process is initiated in rice after flowering, during which a large number of filling materials are transported to the spikelet through the neck panicle node. Therefore, the bleeding intensity of the node can reflect the grain filling condition during the late developmental stages in rice. Based on a previous study that investigated the relationship between the bleeding intensity and filling status in rice grains, the sink activity in weak spikelets was easily interpreted by the bleeding intensity [28–30], therefore, bleeding intensity can be a useful indicator for evaluating the filling condition of weak spikelets in our *Bt* rice study. Zhao [31] showed that the grain plumpness and yield increased in proportion to the amount of material transported into a sink per unit of time and time that this transportation was sustained. This is consistent with the results from our study, as the bleeding intensity was significantly positively correlated with grain yield and setting rate. In addition, the bleeding intensity, setting rate, and yield in T1c-19 (Cry1C*) under different K levels were all remarkably lower than those in the MH63 control, suggesting that the difference of grain yield between the T1c-19 (Cry1C*) *Bt* rice line and the non-transgenic line was not directly related to the K supplied, but was rather mainly due to changes in nutrient transport from the plant to the rice grain.

Potassium (K) is known to affect the transport and allocation of sugar, including glucose, sucrose, starch, et al. [17, 32]. In addition, K is responsible for activating the starch synthetase. And the NSC indicated soluble sugar and starch in the experiment. Thus, the content and transportation condition of NSC in rice were considered to evaluate the influence of K. The NSC stored in the stem sheath before the heading stage is most likely the main source of the filling material during the filling period, and plays an important role in rice yield formation. Several researchers have suggested that the NSC content changes from the heading to maturing stages, indicating that NSC apparent efflux is a key factor contributing to high grain setting rates [33–37]. From Table 1, it was evident that only the setting rate differed significantly and no significant difference was found on 1000 grain weight between the two lines. From Table 5, it was also obvious that 1000 grain weight was not correlating with any vascular characters studied. Hence it can be inferred that the seed set was less in transgenic line compared with seed filling. However, the analysis of NSC may not give sufficient leads as it appears that current photosynthesis also contributing towards better yield realization.

The morphological structure of the neck panicle transportation tissue can directly affect the transfer of NSC from the stem sheaths to the grain, which generates a potentially large sink and fluency flow in rice [38–41]. Significant positive correlations between the neck panicle vascular bundle number in rice and the transportation of assimilation substances synthetized in the stem sheath, number of spikelets, and number of spikelets per panicle have been reported previously [42, 43]. In addition, the number of small vascular bundles and the diameter of the neck panicle was reported to be positively related to the NSC apparent efflux in the stem sheath, which directly affected grain yield [44]. Thick neck panicles possess a large culm wall area that contains more and larger vascular bundles, thereby providing a more efficient transfer environment [38]. In this study, compared to the non-*Bt* rice line, T1c-19 (Cry1C*) had a smaller neck panicle diameter and fewer small vascular bundles, which collectively influence the transportation of NSC in stem sheaths, and contributed to a lower setting rate and grain yield.

## Conclusions

Based on the results, differences in yield performance, flow characters and K distribution were observed between T1c-19 (Cry1C*) and its wildtype. Moreover, there were positive correlations among grain yield, setting rate and several flow characters indicators. However, the source of this variance is not yet clear, although it could be derived from the interference of exogenous and endogenous genes expressed in the transgenic rice, somatic variation during transformation, or the bio-burden of the exogenous genes on energy in crops. Thus, the hereditary cause of the observed character changes needs to be further explored.

## Supporting information

S1 Fig The picture of the potted transgenic and non-transgenic rice plants. (TIF)

S2 Fig The picture of the potted transgenic and non-transgenic rice panicles. (TIF)
