# Supplementary figures and images for: Phloem transport capacity of transgenic rice T1c-19 (Cry1C*) under several potassium fertilizer levels
Source: PLoS One. 2018 Mar 29;13(3):e0195058. doi: 10.1371/journal.pone.0195058 (PMC5875849; doi:10.1371/journal.pone.0195058)

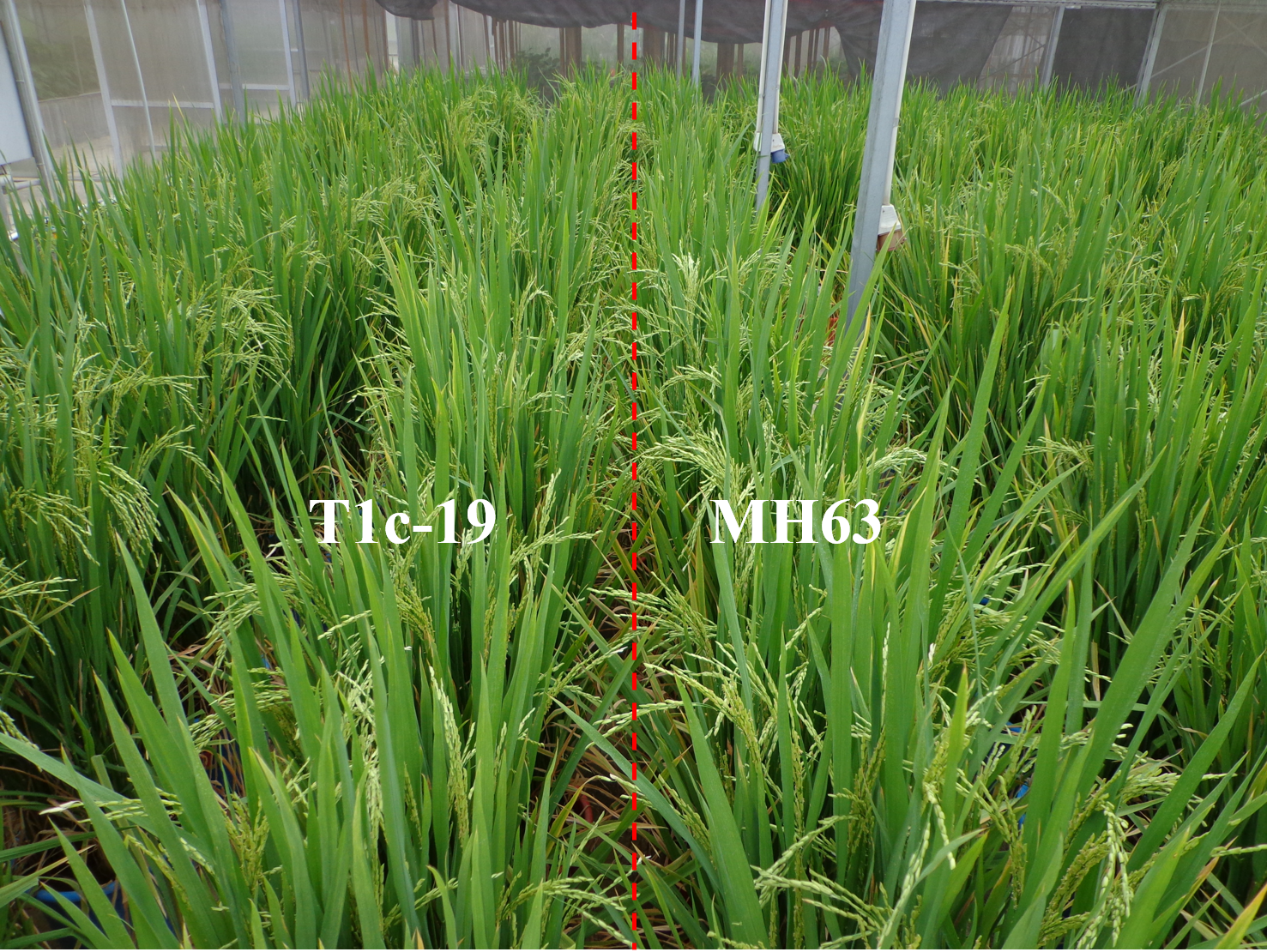

Supplement: S1 Fig — (TIF) [file pone.0195058.s001.tif]

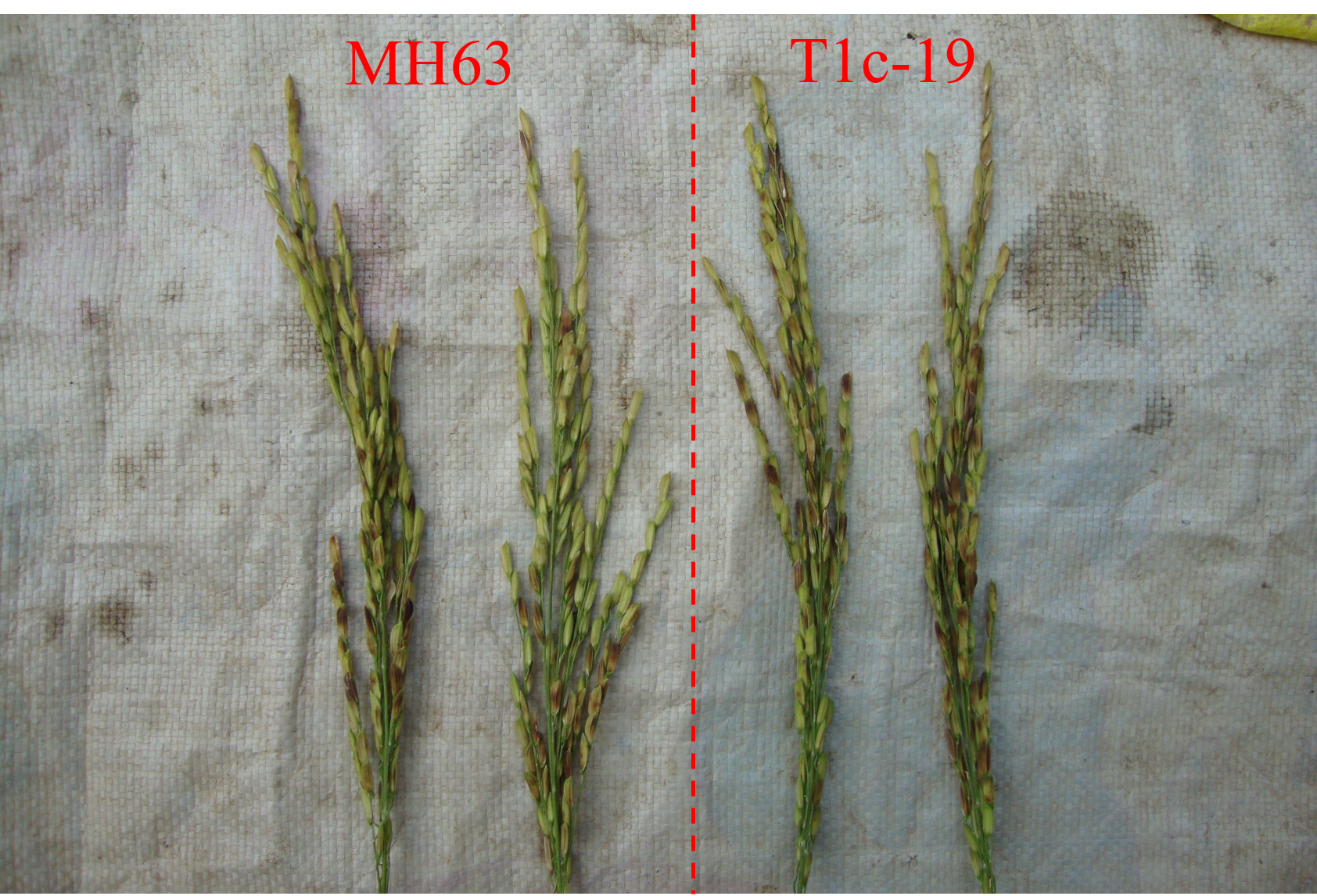

Supplement: S2 Fig — (TIF) [file pone.0195058.s002.tif]
